# Viral infectivity in paediatric SARS-CoV-2 clinical samples does not vary by age

**DOI:** 10.1099/acmi.0.000547.v4

**Published:** 2023-05-26

**Authors:** Madaline M. Schmidt, Hannah W. Despres, David J. Shirley, Michael E. Bose, Kate C. McCaul, Jessica W. Crothers, Kelly J. Henrickson, Benjamin Lee, Emily A. Bruce

**Affiliations:** ^1^​ Department of Microbiology and Molecular Genetics, Robert Larner, MD College of Medicine, University of Vermont, Burlington, VT, USA; ^2^​ Data Science Department, Faraday, Inc., Burlington, VT, USA; ^3^​ Department of Pediatrics, Medical College of Wisconsin, Milwaukee, WI, USA; ^4^​ Department of Pathology and Laboratory Medicine, Robert Larner, MD College of Medicine, University of Vermont, Burlington, VT, USA; ^5^​ Department of Pediatrics, Robert Larner, MD College of Medicine, University of Vermont, Burlington, VT, USA

**Keywords:** SARS-CoV-2, paediatric, viral infectivity, clinical isolates

## Abstract

At the start of the severe acute respiratory syndrome coronavirus 2 (SARS-CoV-2) pandemic, there was much uncertainty about the role of children in infection and transmission dynamics. Through the course of the pandemic, it became clear that children were susceptible to SARS-CoV-2 infection, although they were experiencing a notable lack of severe disease outcomes as compared to the adult population. This trend held true with the emergence of new SARS-CoV-2 variants, even in paediatric populations that were ineligible to be vaccinated. The difference in disease outcomes has prompted questions about the virological features of SARS-CoV-2 infection in this population. In order to determine if there was any difference in the infectivity of the virus produced by children with coronavirus disease 2019 (COVID-19), we compared viral RNA levels (clinical RT-qPCR *C*_T_) and infectious virus titres from 144 SARS-CoV-2-positive clinical samples collected from children aged 0 to 18 years old. We found that age had no impact on the infectiousness of SARS-CoV-2 within our cohort, with children of all ages able to produce high levels of infectious virus.

## Data Summary

The code and dataset (clinical RT-qPCR and infectious viral litretitres) are available at https://github.com/emilybrucelab.

## Introduction

During the early months of the severe acute respiratory syndrome coronavirus 2 (SARS-CoV-2) pandemic, notable uncertainty emerged regarding the role of children in transmission dynamics [1]. With time, it became clearer that children were susceptible to infection with SARS-CoV-2, but that the vast majority of children experienced mild symptoms with lower incidence of severe disease [2]. This pattern remained consistent despite the later emergence of SARS-CoV-2 variants, including delta and omicron, even among children <5 who were ineligible for vaccination [3]. The relative lack of severe disease in the paediatric population raised questions regarding viral kinetics and infectivity in children versus adults.

We hypothesized that unique virological features (i.e. defective interfering genomes or other changes in particle infectivity) in children could explain this apparent decrease in symptoms and transmissibility early in the pandemic. The infectious virus to viral RNA ratio varies greatly in RNA viruses, including SARS-CoV-2, which has a ratio of 10^3^ : 1 to 10^6^ : 1 [4]. This high ratio means that most genome copies are associated with non-infectious particles, thus leaving room for potential changes in the ratio between infectious virus and viral RNA across different age groups. Changes in this ratio have been observed between SARS-CoV-2 variants; both the epsilon and delta variants had significantly higher infectious virus to viral RNA ratios than the alpha variant [5]. Children have been found to produce high levels of viral RNA, but early in the pandemic infectious virus titre was not commonly measured. Plaque or focus assays that directly measure the number of infectious virions are the gold standard for determining viral load. However, they require inoculating a susceptible cell line with serial dilutions of the sample of interest, allowing virus to infect cells under a semi-solid overlay, and detection of individual ‘foci’ of infection by the viral-specific antibodies or the presence of cytopathic effect. Due to the challenges posed by measurement of infectious viral titres (including the requirement for BSL-3 containment), the majority of work examining viral loads in clinical samples has measured viral RNA levels, as determined by RT-qPCR cycle threshold (*C*_T_). A previous study using this technique reported no differences in viral RNA load in adults and children, when controlling for the presence of symptoms [6]. A second study reported both RNA viral load and level of infectious virus using a semi-quantitative method (TCID_50_) in paediatric clinical samples, which revealed that neither age nor disease severity impacted on viral load [7]. In contrast, however, others have found that children have decreased levels of viral RNA, lower infectious virus titres as measured by TCID_50_ and are less likely to have virus successfully isolate in cell culture as compared to adults [8], although paediatric delta variant infections increase the culture-positive viral titres to comparable levels to adults [9]. Other work indicates that ancestral SARS-CoV-2 replicates less efficiently in both children and paediatric versus adult nasal epithelial cells, a defect that omicron was able to abolish [10]. Finally, we and others have demonstrated a dynamic relationship between *C*_T_ values and infectious viral titres with potential for significant discrepancies and a ratio dependent on both viral and host factors [5], but this work did not include children [11].

Therefore, to further understand SARS-CoV-2 infection in children, we investigated the ratio of infectious virus titre to RNA viral load in children aged 0 to <18 years old. We hypothesized that the ratio of infectious virus to RNA viral load would be positively associated with age.

## Methods

### Sample selection

Banked SARS-CoV-2-positive nasopharyngeal specimens from children aged 0 to <18 years old collected and stored at Children’s Wisconsin, Milwaukee, Wisconsin, USA between 14 September 2020 and 17 May 2021 were identified. Deidentified samples were binned into four age groups (<1, 1–5, 6–11 and 12–17) and stratified by clinical *C*_T_ value (<20, 20–24, 25–29 and 30–34) to select a sample of children representing the full spectrum of both ages and *C*_T_ values (due to the deidentified nature of the samples, information on biological sex and other metadata was not available).

### RNA extractions and RT-PCR

Total nucleic acid was extracted on the NucliSENS easyMAG or EMAG automated extraction instruments (bioMérieux). SARS-CoV-2 RNA was detected using previously published primers/probes for the SARS-CoV-2 E gene (Sarbeco [12]) on the 7500 Fast Real-Time PCR System or QuantStudio 7 Pro platforms.

### Viral titre

All SARS-CoV-2 viral titring was conducted at the University of Vermont BSL-3 facility under an approved IBC protocol. Clinical samples were titred using a microfocus forming assay on VeroE6-TMPRSS2 cells (Japanese Cancer Research Resources Bank #JCRB1819). Cells were seeded 24 h before infection, 60 000 cells/well. Samples were serially diluted using 10-fold dilutions. All samples were titred in technical duplicate, except for the neat dilution due to insufficient sample volume. Cells were infected for 1 h at 37 °C, and then overlaid with 1.2 % methylcellulose in Dulbecco's Modified Eagle Medium (DMEM) (Gibco cat. 11965084) and incubated for 24 h at 37 °C, 5 % CO_2_. Cells were fixed using 4 % formaldehyde in phosphate-buffered saline (PBS). After fixation, cells were permeabilized using 0.01 % Triton X-100 in PBS for 15 min and then incubated in a primary, cross-reactive rabbit anti-SARS-CoV N monoclonal antibody (Sino Biological 40143R001100) at 1 : 20 000 dilution. Cells were washed in PBS and then incubated in a peroxidase-labelled goat-anti-rabbit antibody (Seracare 5220–0337) at 1 : 4000, followed by the peroxidase substrate (SeraCare, cat. #5510–0030). Foci were imaged using a BioTek Cytation instrument and counted manually. Samples were titred in a blinded fashion; personnel performing BSL-3 titring did not have access to sample *C*_T_s.

### Statistical analysis

Viral titres were log-transformed for analysis. Linear regression was used to predict log titre as a function of *C*_T_, fitting separate models without age and to control for continuous and categorical age effects, and no data points were excluded from analysis. Models were compared by F test. Data were analysed and plotted with R.

## Results

**Fig. 1.**
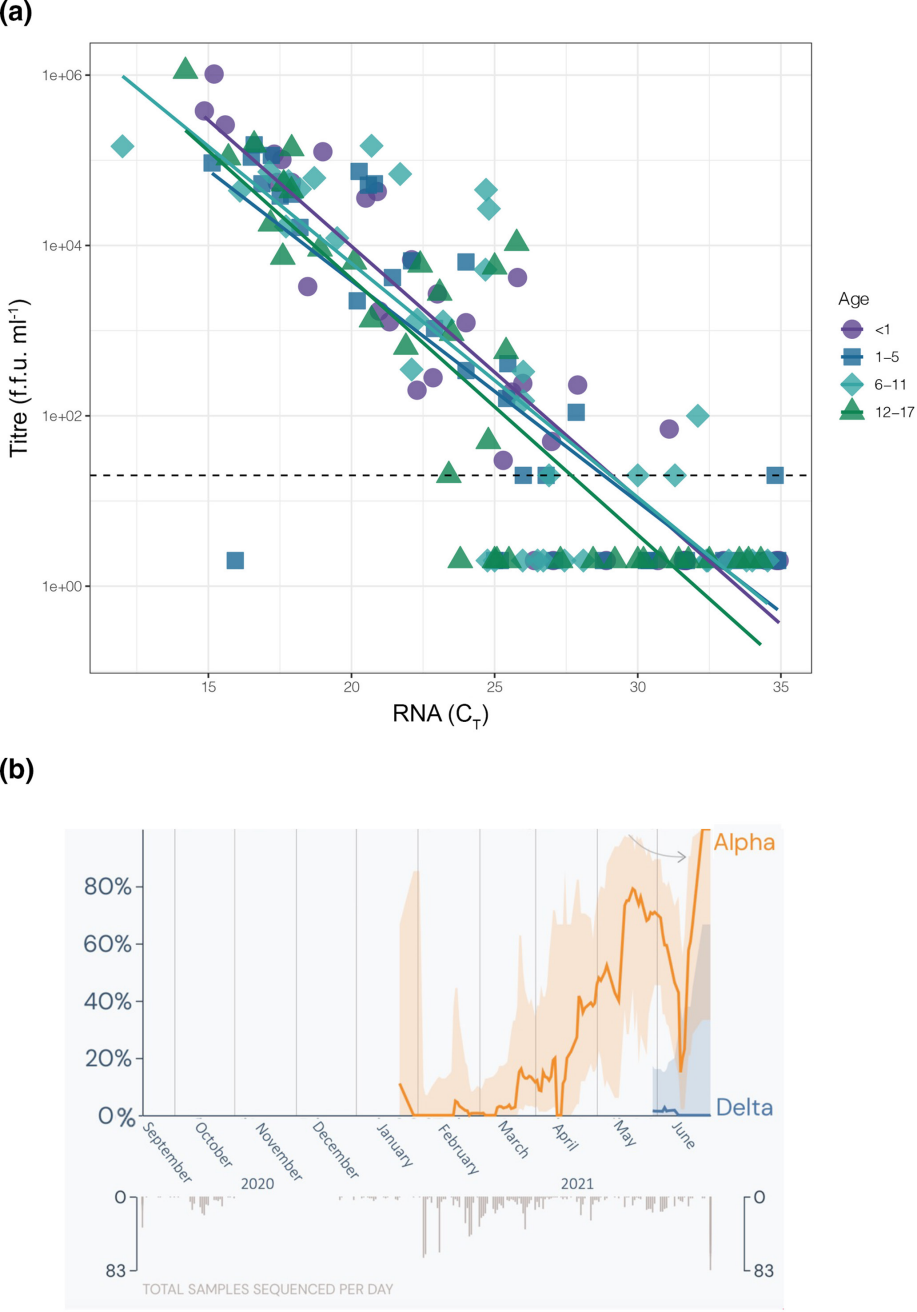
SARS-CoV-2 viral infectivity does not vary by age in a paediatric population. (a) A set of 144 clinical samples from children infected with SARS-CoV-2 was used to examine the relationship between infectious virus titre and RNA viral load as a function of patient age. Individual specimen measurements of E gene RNA levels (*C*_T_) on the *x*-axis are plotted against viral titre, as measured in focus forming units (f.f.u. ml^−1^) on the *y*-axis. Dashed line indicates the limit of detection for infectious titre (20 f.f.u. ml^−1^). Samples for which we could not measure a viral titre were assigned fixed values of one-tenth the limit of detection (2 f.f.u. ml^−1^). Lines of best fit were generated by linear regression on log-transformed titre data as a function of *C*_T_ and age group. Symbol and colour indicate age group (<1, purple circle; 1–5, blue square; 6–11, turquoise diamond; 12–17, green triangle). (**b**) During the time of sample acquisition, epidemiological data about SARS-CoV-2 variants were obtained in Milwaukee, Wisconsin, USA. Sequencing prior to January 2021 indicates the presence of ancestral (pre-VOC) SARS-CoV-2, and post January 2021 through June 2021 sequencing primarily detects the alpha variant. The epidemiological data collected include both adult and paediatric samples and are subject to sampling bias. The number of samples sequenced per day varied from 0 to 83. The solid line indicates the 7 day rolling average of the percentage of sequences with mutations, and the shaded area represents the 95 % confidence interval. Sequencing data adapted from outbreak.info, April 2021 [14].

*n*=144 clinical specimens were selected to determine the relationship between the infectivity of SARS-CoV-2 in paediatric samples and RNA viral load. As expected, higher RNA viral load generally correlated with higher infectious virus titre, although as reported previously this ratio was somewhat variable (Fig. 1a) [5, 11]. In linear regression, the relationship between infectious viral titre and *C*_T_ was not significantly modified by age (*P*=0.156) or age group (*P*=0.355 overall by F test). These data indicate that there is no difference in the infectiousness of SARS-CoV-2 produced by children, regardless of age.

## Discussion

Consistent with previous findings, we found no significant differences in the relationship between SARS-CoV-2 infectious virus titre and RNA viral load in children across the paediatric age spectrum [6, 7]. Our findings suggest equal levels of viral infectivity in young children and adolescents with similar RNA viral loads, thus other factors are contributing to differential disease severity in this population. Interestingly, while we and others have found equal levels of viral infectivity across paediatric age groups, it has been found that age does influence severity of disease within the paediatric population. In one cohort, asymptomatic patients and those with mild disease were significantly younger than those hospitalized, suggesting that age-related factors other than viral infectivity are playing a role in disease manifestation [7]. This could include differences in immune response (i.e. heavier reliance on the innate immune response), fewer comorbidities, or even fewer viral entry receptors than adults [13]. The limitations of this study include lack of access to viral sequencing and individual-level metadata, which could reveal differences in infectivity as a result of viral genetic background, days post-symptom onset, host immune status and vaccination status. Furthermore, as sample collection took place at a children’s hospital there was no direct comparison with adult samples, although we did include samples in older teens who would closely resemble adults biologically. While our results are in agreement with several recent studies, replicating prior work in a novel cohort with independent methodologies is an important step in establishing a clear understanding of SARS-CoV-2 infection dynamics in children. While we only had access to samples early in the pandemic (during the circulation of alpha and prior to the appearance of variants of concern; Fig. 1b), future studies investigating the viral infectivity in paediatric patients during waves of delta, omicron and future variants would strengthen our understanding of the dynamics of SARS-CoV-2 infection in the paediatric population. Regardless, the finding that children of all ages with confirmed SARS-CoV-2 infection are capable of producing high levels of replication-competent virus should be balanced against the finding that there was no age-dependent effect, when designing public health policy-based interventions.
